# Opicapone to Treat Early Wearing‐off in Parkinson's Disease Patients: The Korean ADOPTION Trial

**DOI:** 10.1002/mdc3.14030

**Published:** 2024-04-09

**Authors:** Jee‐Young Lee, Hyeo‐il Ma, Joaquim J. Ferreira, José‐Francisco Rocha, Young Hee Sung, In‐Uk Song, Tae‐Beom Ahn, Do Young Kwon, Sang‐Myung Cheon, Jong‐Min Kim, Chong Sik Lee, Phil Hyu Lee, Jeong‐Ho Park, Jae‐Hyeok Lee, Mee Young Park, Sang Jin Kim, Jong Sam Baik, Seong‐Min Choi, Hae‐Won Shin, Ho‐Won Lee, Suk Yun Kang, Beomseok Jeon

**Affiliations:** ^1^ Department of Neurology SMG‐SNU Boramae Medical Center Seoul Korea; ^2^ Department of Neurology Hallym University Sacred Heart Hospital Anyang Korea; ^3^ IMM – Instituto de Medicina Molecular João Lobo Antunes, Faculdade de Medicina, Universidade de Lisboa Lisbon Portugal; ^4^ CNS – Campus Neurológico Torres Vedras Portugal; ^5^ BIAL – Portela & C^a^ S.A Coronado Portugal; ^6^ Gachon University Gil Medical Center Incheon Korea; ^7^ The Catholic University of Korea Incheon St. Mary's Hospital Incheon Korea; ^8^ Kyung Hee University Medical Center Seoul Korea; ^9^ Korea University Ansan Hospital Ansan Korea; ^10^ Dong‐A University Hospital Busan Korea; ^11^ Seoul National University Bundang Hospital Seongnam Korea; ^12^ Asan Medical Center Seoul Korea; ^13^ Severance Hospital Seoul Korea; ^14^ Soon Chun Hyang University Hospital Bucheon Bucheon Korea; ^15^ Pusan National University Yangsan Hospital Yangsan Korea; ^16^ Yeungnam University Medical Center Daegu Korea; ^17^ Inje University Busan Paik Hospital Busan Korea; ^18^ Inje University Sanggye Paik Hospital Seoul Korea; ^19^ Chonnam National University Hospital, Chonnam National University Medical School Gwangju Korea; ^20^ Chung‐ang University Hospital Seoul Korea; ^21^ Kyungpook National University Chilgok Hospital Daegu Korea; ^22^ Dongtan Sacred Heart Hospital Hallym University College of Medicine Hwaseong Korea; ^23^ Department of Neurology Seoul National University Hospital Seoul Korea

**Keywords:** levodopa, opicapone, Parkinson's disease, wearing off

## Abstract

**Background:**

Increasing levodopa (L‐dopa)/dopa decarboxylase inhibitor (DDCI) daily dose or adding a catechol‐O‐methyltransferase (COMT) inhibitor to levodopa/DDCI therapy are strategies used to manage wearing‐off symptoms in Parkinson's disease (PD) patients.

**Objectives:**

To evaluate the COMT inhibitor opicapone versus an additional dose of levodopa to treat early wearing‐off in PD patients.

**Methods:**

ADOPTION was a randomized, parallel‐group, open‐label, Phase 4 study conducted in Korea. At baseline, eligible patients were randomized (1:1) to opicapone 50 mg (n = 87) or L‐dopa 100 mg (n = 81) (added to current L‐dopa/DDCI therapy) for 4 weeks. The main efficacy endpoint was change from baseline to end of study in absolute *off* time. Other endpoints included changes in *on* time, in Movement Disorder Society‐Unified Parkinson's Disease Rating Scale and 8‐item PD Questionnaire scores, and the Clinical and Patient Global Impression of Improvement/Change.

**Results:**

The adjusted mean in absolute *off* time was significantly greater for opicapone 50 mg than for L‐dopa 100 mg (−62.1 vs. −16.7 minutes; *P* = 0.0015). Opicapone‐treated patients also reported a greater reduction in the percentage of *off* time (*P* = 0.0015), a greater increase in absolute *on* time (*P* = 0.0338) and a greater increase in the percentage of *on* time (*P* = 0.0015). There were no significant differences in other secondary endpoints. The L‐dopa equivalent daily dose was significantly higher in the opicapone group (750.9 vs. 690.0 mg; *P* = 0.0247), when a 0.5 conversion factor is applied.

**Conclusions:**

Opicapone 50 mg was more effective than an additional 100 mg L‐dopa dose at decreasing *off* time in patients with PD and early wearing‐off.

## Introduction

In the early stages of Parkinson's disease (PD), levodopa (L‐dopa)/dopa decarboxylase inhibitor (DDCI) therapy is usually associated with significant improvements in motor disability. The first few years of L‐dopa therapy are often referred to as the “honeymoon period” because patients generally enjoy sustained symptomatic relief with minimal side effects.^1^ This is because, in the early stages of the disease, presynaptic neurons maintain their capacity to store and slowly release dopamine generated from exogenous L‐dopa and are therefore able to buffer any variability in plasma L‐dopa levels.^2^, ^3^, ^4^, ^5^ However, as the disease progresses, nigrostriatal dopaminergic neurons continue to degenerate and presynaptic neurons lose their ability to store dopamine, and thus their initial buffering capacity.^6^ The response to L‐dopa therefore progressively fluctuates mirroring its pulsatile plasma levels, which results in wearing‐off, the earliest and most frequent complication in patients with PD.^4^, ^7^ Although wearing‐off symptoms are frequent, they are often not adequately recognized and, particularly for non‐motor components, not well defined because they are not closely related to a reduction in motor performance.^8^ Common therapeutic strategies to manage early wearing‐off symptoms include increasing the total daily dose of L‐dopa/DDCI and/or fractioning the total dose in smaller, more frequent doses.^9^, ^10^ However, clinical experience suggests that these conventional strategies are short‐term solutions and are associated with an increased risk of dyskinesia and/or suboptimal exposure for patients^10^ and might therefore not represent the optimal approach to treat wearing‐off. Furthermore, these strategies do not address the pulsatile nature and short half‐life of L‐dopa. An alternative pharmacological approach is to optimize L‐dopa delivery by administering L‐dopa/DDCI with catechol‐*O*‐methyltransferase (COMT) inhibitors that increase the plasma half‐life of L‐dopa, thus extending the duration of its clinical effect.^10^, ^11^, ^12^, ^13^ The efficacy of opicapone, a third generation, once‐daily COMT inhibitor, is well established in patients with PD and motor fluctuations.^14^, ^15^ There is also evidence for enhanced efficacy of opicapone in patients who were earlier versus later in their disease course and L‐dopa treatment pathway, suggesting that it might be an effective early adjunct to L‐dopa.^16^ However, the clinical efficacy of opicapone in the management of early wearing‐off as a putative alternative to conventional L‐dopa strategies is still unknown. A recent exploratory Phase II study showed that, combining opicapone 50 mg with a 100 mg lower daily dose of L‐dopa/carbidopa regimen still provides higher L‐dopa bioavailability, avoidance of trough levels and, despite the lower L‐dopa dose, a decrease in *off* time.^12^ Thus, the aim of this study was to evaluate the effects of opicapone versus an additional 100 mg dose of L‐dopa/DDCI to treat early wearing‐off in patients with PD.

## Methods

The e**A**rly L‐**D**opa with **O**picapone in **P**arkinson's pa**T**ients w**I**th mot**O**r fluctuatio**N**s (ADOPTION) study was a randomized, parallel‐group, multicenter, prospective, open‐label, exploratory, Phase 4 study conducted from June 2021 to August 2022 in Korea.

### Study Population

Male or female patients aged ≥30 years were eligible if they had a diagnosis of idiopathic PD and a modified Hoehn & Yahr stage of 1–3 (at *on* state). Patients had to have been treated with L‐dopa/DDCI, up to a maximum of 600 mg given 3–4 times daily for ≥4 weeks prior to study entry and had to demonstrate signs of wearing‐off (average of total daily *off* time ≥1 hour) for ≥4 weeks but <2 years. Key exclusion criteria included non‐idiopathic PD, severe and/or unpredictable *off* periods or an average total daily *off* time >5 hours while awake. Previous or planned surgery for deep brain stimulation and treatment with monoamine oxidase inhibitors (except for selegiline ≤10 mg/day oral and ≤1.25 mg/day buccal absorption formulations, rasagiline ≤1 mg/day and safinamide ≤100 mg/day), entacapone, tolcapone and anti‐emetics with anti‐dopaminergic action (except domperidone) were prohibited during the study (withdrawn ≥4 weeks before screening).

### Study Design

The study design is illustrated in Fig. S1. Following screening, eligible patients were randomized (1:1) to opicapone 50 mg or L‐dopa/DDCI 100 mg (referred to as L‐dopa 100 mg) for 4 weeks in addition to their current L‐dopa/DDCI therapy. Opicapone was administered once daily at bedtime 1 hour after L‐dopa/DDCI intake, and L‐dopa 100 mg was administered either as a 100‐mg full intake or two 50‐mg intakes distributed across any of the previous intakes. During the first 2 weeks of study treatment, the total daily dose of L‐dopa/DDCI (excluding the additional 100 mg dose of L‐dopa) or the intake intervals could be adjusted in case of dopaminergic adverse events (AEs). For the last 2 weeks of the study, the daily dose of L‐dopa/DDCI was to be maintained stable.

### Study Assessments

The main efficacy endpoint was the change from baseline to the end of study treatment in absolute *off* time, as assessed by daily paper patient diaries.^17^ Other efficacy endpoints were the change from baseline to the end of study treatment in the proportion of patients (ie, responders) achieving at least a 1‐hour reduction in absolute *off* time and the change from baseline to the end of study treatment in the proportion of patients achieving at least a 1‐hour increase in absolute total *on* time. Other diary‐based efficacy variables were changes from baseline in the percentage of *off* time, in absolute *on* time, in the percentage of *on* time, and in *on* time with and without dyskinesia. *Off* and *on* times were calculated as the mean time reported in the 3 preceding diary days, or the mean of available days if fewer than 3 days were recorded. Percentages of *off* and *on* time were calculated as the sum in mins from 30‐min periods classified as *off* or *on* state divided by the total time awake. Scale‐based efficacy variables were the change from baseline in the Movement Disorder Society‐Sponsored Revision of the Unified Parkinson's Disease Rating Scale (MDS‐UPDRS),^18^ 8‐item Parkinson's Disease Questionnaire (PDQ‐8),^19^ and the Clinical Global Impression of Improvement (CGI‐I) and Patient Global Impression of Change (PGI‐C). Levodopa equivalent daily dose (LEDD) was calculated for monoamine oxidase B inhibitors and dopamine agonists using the existing conversion factors.^20^ Safety was assessed for the duration of the study and up to 2 weeks after the end of study by evaluating AEs, serious AEs, drug‐related AEs and AEs leading to discontinuation.

### Statistical Analyses

The study was deemed to be exploratory by nature as no hypothesis‐testing was created due to non‐existing data on the magnitude of effect of L‐dopa 100 mg. Still, even if exploratory with no formal sample size calculation, it was deemed that approximately 100 patients per arm would be a sufficient number to allow a robust analysis and interpretation of the outcomes. Population sets were defined as the full analysis set, which included all randomly assigned patients who took at least one dose of study drug and had at least one efficacy assessment after baseline; the per‐protocol set, which included all patients in the full analysis set who did not have any major protocol deviations; and the safety set, which included all patients who received at least one dose of study drug. The efficacy analysis did not follow any hierarchical procedure and we used the intention‐to‐treat approach with the full analysis set because it is the most conservative method to test. All data were primarily summarized using descriptive statistics. Continuous variables were reported as mean, standard deviation (SD), and number of subjects. Categorical variables were reported as counts and percentages. Only patients with available (non‐missing) data for a particular variable were included in the calculation of a percentage. ANCOVA models were used to evaluate the applicable endpoints as independent variables, treatment arm as fixed effect and baseline values as covariate. Adjusted means, standard error (SE), 95% confidence intervals (CI) and *P*‐values for treatment difference will be presented for the effect estimates. The responder rates of change in *off/on*‐time will be compared between the treatment groups using the Chi‐square test.

### Ethical Approval and Consent

The clinical study protocol and the informed consent form were reviewed and approved by the respective independent ethics committee. All patients included in the study signed a patient consent form.

### Data Sharing

The data that support the findings of this study are available from the corresponding author upon reasonable request. Data are not publicly available due to privacy or ethical restrictions.

## Results

### Study Population

Of the initially planned 200 patients, 193 patients were recruited from 19 centers across Korea. Of those, 169 patients were randomized to either opicapone 50 mg (N = 88) or L‐dopa 100 mg (N = 81). One patient did not receive any investigational drug, and therefore the safety analysis set was comprised of 168 patients (opicapone 50 mg, n = 87; L‐dopa 100 mg, n = 81). As three patients did not have efficacy assessments, the full analysis set included 165 patients (opicapone 50 mg, n = 84; L‐dopa 100 mg, n = 81) (Fig. S2). Baseline demographics and disease characteristics were similar between the two treatment arms (Table 1). In the overall population, the mean (SD) age was 64.1 (7.7) years, and the mean duration of PD was 5.3 (3.6) years. The mean (SD) daily *off* time was 3.4 (1.0) hours (~21% of their awake time) and most of *on* time spent at *on* state was without dyskinesia (~87% of total *on* time). The mean baseline L‐dopa daily dose was ~400 mg, mainly distributed over three daily intakes. The majority of patients were taking pramipexole (62.5%) and rasagiline (58.3%) as anti‐PD medication at baseline and the mean (SD) LEDD was 580.8 (179.8) mg in the overall population, 569.8 (181.7) mg in the opicapone 50 mg group and 592.8 (178.0) mg in the L‐dopa 100 mg group (Table 1). During the study, 15.1% of patients in the opicapone 50 mg group and 21.0% in the L‐dopa 100 mg group required L‐dopa dose adjustments (the additional L‐dopa dose of 100 mg was excluded), primarily due to AEs in the opicapone 50 mg group (n = 11), resulting in a decrease in the mean L‐dopa daily dose of ~7 mg in the opicapone 50 mg group (from 402.7 mg/day to 396.0 mg/day) and of ~2 mg in the L‐dopa 100 mg group (from 414.4 mg/day to 412.0 mg/day) (data not shown). At endpoint, the mean LEDD, according to each respective opicapone 50 mg (using a 0.5 conversion factor) and L‐dopa 100 mg conversion factor, was significantly higher for the opicapone 50 mg group (750.9 mg) than for the L‐dopa 100 mg group (690.0 mg, *P* = 0.0247; Table 2); when a conversion factor of 0.33 is used for opicapone, a similar LEDD was reported between the two groups (684.2 vs. 690.0 mg; *P* = not significant) (Table 2). Few patients (~10%) discontinued the study, with the most common reasons being AEs (opicapone 50 mg, n = 4; L‐dopa 100 mg, n = 2) and/or major protocol deviations (opicapone 50 mg, n = 3; L‐dopa 100 mg, n = 3).

**TABLE 1:** Baseline characteristics in the randomized set

	Opicapone 50 mg	L‐dopa 100 mg	*P* Value	Total
	N = 88	N = 81		N = 169
Mean age, year (SD)	64.1 (7.5)	64.2 (8.0)	0.91^a^	64.1 (7.7)
Male, n (%)	39 (44.3)	44 (54.3)	0.19^b^	83 (49.1)
Mean height, cm (SD)	161.0 (9.0)	162.1 (8.7)^c^	0.43^a^	161.5 (8.8)
Mean weight, kg (SD)	62.8 (10.1)	63.7 (10.7)^c^	0.70^d^	63.2 (10.4)
Mean H&Y, stage (SD)	2.0 (0.5)	2.1 (0.6)	0.52^d^	2.0 (0.5)
Mean PD duration, year (SD)	5.0 (3.5)	5.7 (3.8)	0.29^d^	5.3 (3.6)
Mean MDS‐UPDRS, score (SD)^e^				
Part I	6.4 (4.0)	7.3 (4.5)	0.19^d^	6.8 (4.3)
Part II	6.6 (4.7)	8.1 (5.9)	0.15^d^	7.3 (5.4)
Part III	22.0 (10.0)	23.7 (11.5)	0.38^d^	22.8 (10.8)
Part IV	4.0 (1.7)	4.2 (1.9)	0.72^d^	4.1 (1.8)
Total	39.0 (14.5)	43.3 (18.9)	0.24^d^	41.1 (16.9)
Mean PDQ‐8, score (SD)^e^	5.3 (4.3)	5.6 (4.7)	0.75^d^	5.4 (4.5)
Mean daily *off* time, hours (SD)^e^	3.4 (1.1)	3.4 (1.0)	0.84^d^	3.4 (1.0)
Mean total *on* time, hours (SD)^e^	13.0 (1.7)	13.0 (1.5)	0.83^a^	13.0 (1.6)
Mean *on* time without dyskinesia, hours (SD)^e^	11.4 (3.0)	11.3 (3.5)	0.63^d^	11.3 (3.2)
Mean L‐dopa amount at baseline, mg (SD)^f^	402.7 (127.7)	414.4 (120.3)	0.60^d^	408.3 (123.9)
Patients receiving 3 or 4 L‐dopa intakes per day, n (%)				
3 intakes	81 (92.0)	74 (91.4)	0.87^b^	155 (91.7)
4 intakes	7 (8.0)	7 (8.6)	0.87^b^	14 (8.3)
Patients receiving MAO‐Bi and/or DA, *n* (%)	74 (84.1)	70 (86.4)	0.67^b^	144 (85.2)
DA	55 (65.5)	60 (74.1)	0.23^b^	115 (68.0)
Pramipexole	43 (58.1)	47 (67.1)	0.26^b^	90 (62.5)
Ropinirole	18 (24.3)	15 (21.4)	0.68^b^	33 (22.9)
MAO‐Bi	47 (56.0)	42 (51.9)	0.60^b^	89 (52.7)
Rasagiline	46 (62.2)	38 (54.3)	0.34^b^	84 (58.3)
Selegiline	1 (1.4)	3 (4.3)	0.36^g^	4 (2.8)
Safinamide	1 (1.4)	1 (1.4)	1.00^g^	2 (1.4)
LEDD^h^, mg (SD)	569.8 (181.7)	592.8 (178.0)	0.52^d^	580.8 (179.8)

Abbreviations: DA, dopamine agonists; H&Y, Hoehn and Yahr; LEDD, levodopa equivalent daily dose; MAO‐Bi, monoamine oxidase B inhibitors; MDS‐UDPRS, Movement Disorder Society‐Sponsored Revision of the Unified Parkinson's Disease Rating Scale; PD, Parkinson's disease; PDQ, Parkinson's disease questionnaire; SD, standard deviation.

^a^ Two‐sample t‐test.

^b^ Chi‐square test.

^c^ 80 patients.

^d^ Wilcoxon's rank sum test.

^e^ Full Analysis Set.

^f^ Safety Set.

^g^ Fisher's‐exact test.

^h^ Calculated for L‐dopa, MAO‐Bi and DA only.

**TABLE 2:** Efficacy outcomes at end of study treatment in the full analysis set

	Opicapone 50 mg	L‐dopa 100 mg
	n = 84	n = 81
**OFF‐time** (min)		
Adjusted mean ± SE change from baseline	−62.1 ± 9.8	−16.7 ± 10.0
Mean difference vs. L‐dopa 100 mg (95% CI)	−45.4 (−73.1, −17.6)	
*P*‐value for opicapone 50 mg vs. L‐dopa 100 mg	**0.0015**	
**Percent OFF‐time** (%)		
Adjusted mean ± SE change from baseline	−6.5 ± 1.0	−2.0 ± 1.0
Mean difference vs. L‐dopa 100 mg (95% CI)	−4.5 (−7.3, −1.7)	
*P*‐value for opicapone 50 mg vs. L‐dopa 100 mg	**0.0015**	
**OFF‐time responder rate** (reduction of ≥1 hour); n (%)	44 (52.4)	35 (43.2)
*P*‐value for opicapone 50 mg vs. L‐dopa 100 mg	0.2384	
**Total ON‐time** (min)		
Adjusted mean ± SE change from baseline	70.2 ± 11.3	35.6 ± 11.5
Mean difference vs. L‐dopa 100 mg (95% CI)	34.5 (2.7, 66.4)	
*P*‐value for opicapone 50 mg vs. L‐dopa 100 mg	**0.0338**	
**Percent Total ON‐time** (%)		
Adjusted mean ± SE change from baseline	6.5 ± 1.0	2.0 ± 1.0
Mean difference vs. L‐dopa 100 mg (95% CI)	4.5 (1.7, 7.3)	
*P*‐value for opicapone 50 mg vs. L‐dopa 100 mg	**0.0015**	
**ON‐time responder rate** (increase of ≥1 hour); n (%)	47 (56.0)	39 (48.2)
*P*‐value for opicapone 50 mg vs. L‐dopa 100 mg	0.3158	
**ON‐time without dyskinesia** (min)		
Adjusted mean ± SE change from baseline	64.8 ± 13.4	56.6 ± 13.6
Mean difference vs. L‐dopa 100 mg (95% CI)	8.2 (−29.5, 45.9)	
*P*‐value for opicapone 50 mg vs. L‐dopa 100 mg	0.6679	
**ON‐time with troublesome dyskinesia** (min)		
Adjusted mean ± SE change from baseline	−5.8 ± 8.6	−2.3 ± 8.7
Mean difference vs. L‐dopa 100 mg (95% CI)	−3.5 (−27.7, 20.6)	
*P*‐value for opicapone 50 mg vs. L‐dopa 100 mg	0.7721	
**MDS‐UPDRS scores**		
**Part I**		
Adjusted mean ± SE change from baseline	−0.2 ± 0.3	−0.7 ± 0.3
Mean difference vs. L‐dopa 100 mg (95% CI)	0.5 (−0.3, 1.3)	
*P*‐value for opicapone 50 mg vs. L‐dopa 100 mg	0.2350	
**Part II**		
Adjusted mean ± SE change from baseline	−1.2 ± 0.4	−0.4 ± 0.3
Mean difference vs. L‐dopa 100 mg (95% CI)	−0.8 (−1.8, 0.1)	
*P*‐value for opicapone 50 mg vs. L‐dopa 100 mg	0.0914	
**Part III**		
Adjusted mean ± SE change from baseline	−3.4 ± 0.7	−2.5 ± 0.7
Mean difference vs. L‐dopa 100 mg (95% CI)	−0.9 (−2.9, 1.1)	
*P*‐value for opicapone 50 mg vs. L‐dopa 100 mg	0.3591	
**Part IV**		
Adjusted mean ± SE change from baseline	−0.8 ± 0.2	−0.6 ± 0.2
Mean difference vs. L‐dopa 100 mg (95% CI)	−0.2 (−0.7, 0.3)	
*P*‐value for opicapone 50 mg vs. L‐dopa 100 mg	0.3702	
**Total**		
Adjusted mean ± SE change from baseline	−5.4 ± 1.1	−4.3 ± 1.0
Mean difference vs. L‐dopa 100 mg (95% CI)	−1.1 (−4.0, 1.9)	
*P*‐value for opicapone 50 mg vs. L‐dopa 100 mg	0.4782	
**PDQ‐8 scores**		
Adjusted mean ± SE change from baseline	−2.6 ± 1.3	−1.1 ± 1.2
Mean difference vs. L‐dopa 100 mg (95% CI)	−1.6 (−5.1, 1.9)	
*P*‐value for opicapone 50 mg vs. L‐dopa 100 mg	0.3620	
**CGI‐I**		
Participants with improvement^a^, n (%)	62 (80.5%)	54 (67.5%)
**PGI‐C**		
Participants with improvement^b^, n (%)	59 (77.6%)	48 (60.0%)
**LEDD using a conversion factor of 0.5**		
Mean (SD) at end of study	750.9 (216.7)	690.0 (179.3)
*P*‐value for opicapone 50 mg vs. L‐dopa 100 mg	**0.0247** ^c^	
**LEDD using a conversion factor of 0.33**		
Mean (SD) at end of study	684.2 (199.9)	690.0 (179.3)
*P*‐value for opicapone 50 mg vs. L‐dopa 100 mg	0.9260^c^	

*Note*: Significant *P*‐values are in bold.

Abbreviations: CI, confidence interval; CGI‐I, Clinical Global Impressions of Improvement; L‐dopa, levodopa; LEDD, levodopa equivalent daily dose; MDS‐UDPRS; Movement Disorder Society‐Sponsored Revision of the Unified Parkinson's Disease Rating Scale; PDQ, Parkinson's disease questionnaire; PGI‐C, Patient's Global Impression of Change; SE, standard error.

^a^ Opicapone 50 mg, n = 77; L‐dopa 100 mg, n = 80.

^b^ Opicapone 50 mg, n = 76; L‐dopa 100 mg, n = 80.

^c^ Wilcoxon's rank sum test.

### Efficacy

**Figure 1:**
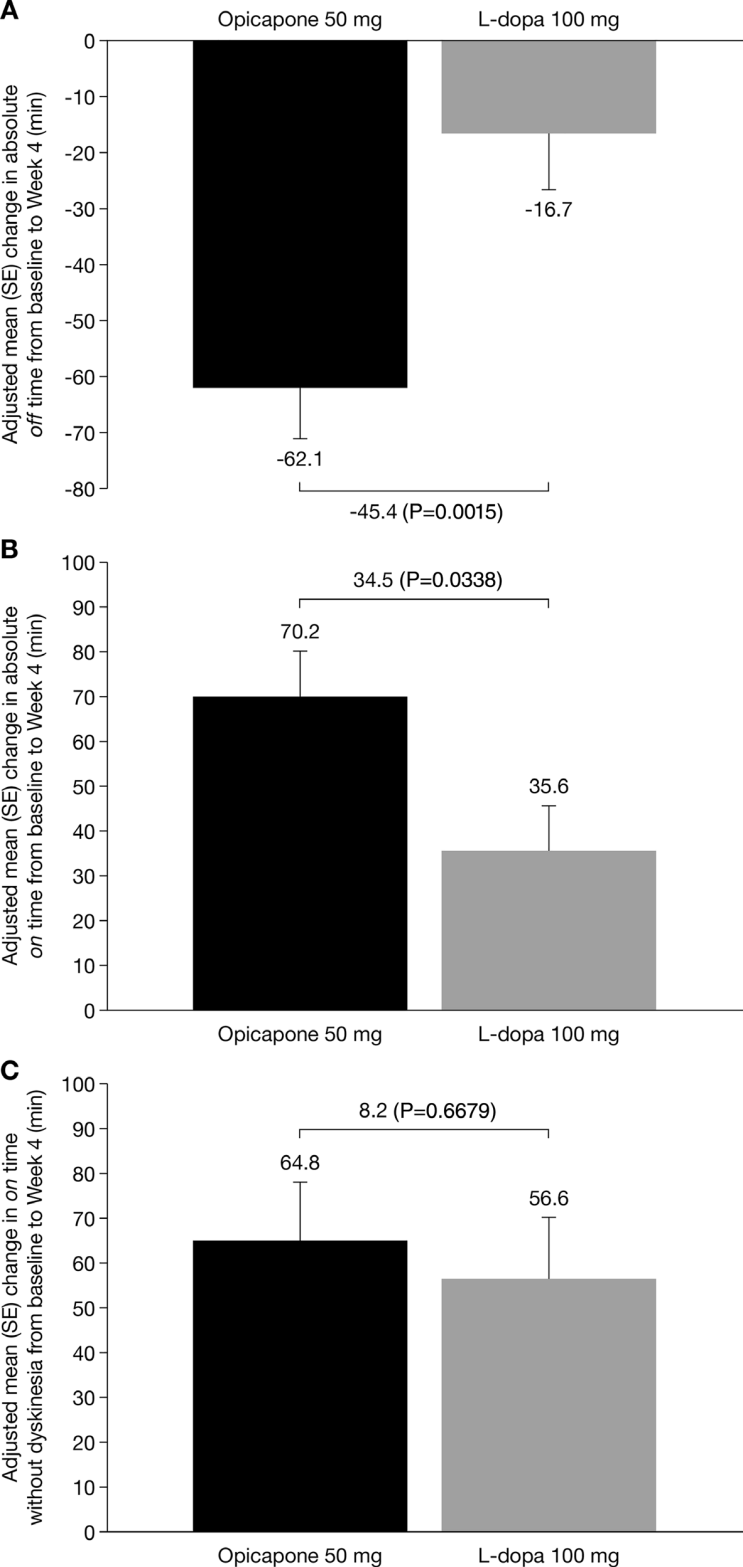
Adjusted mean (SE) change from baseline to end of study treatment in the full analysis set in **A**: absolute *off* time. **B**: absolute *on* time. **C**: absolute *on* time without dyskinesia. L‐dopa, levodopa; SE standard error.

**Figure 2:**
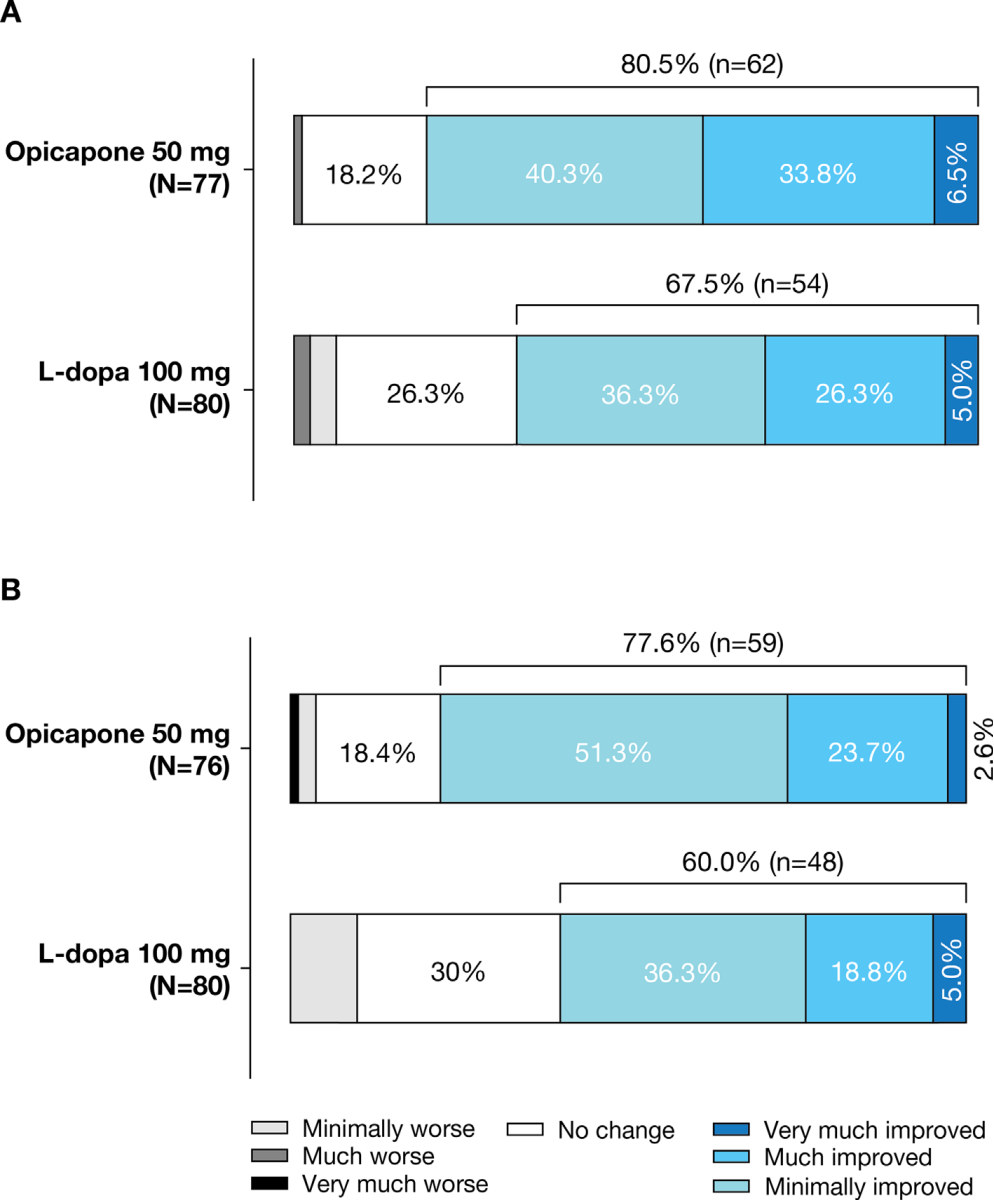
Results in the opicapone 50 mg group and L‐dopa 100 mg group in the full analysis set for **A**: the Clinical Global Impression of Improvement. **B**: the Patient Global Impression of Change. L‐dopa, levodopa.

In the full analysis set, the adjusted mean (SE) change from baseline to end of study treatment in absolute *off* time (main endpoint) was greater for the opicapone 50 mg group than the L‐dopa 100 mg group (−62.1 min [9.8] vs. −16.7 min [10.0]; Fig. 1A, Table 2), resulting in a statistically significant difference of −45.4 mins favoring opicapone 50 mg (95% CI: −73.1, −17.6; *P* = 0.0015; Table 2). Similar results were noted in the per‐protocol set (data not shown). The proportion of patients with a reduction in *off* time of at least 1 hour or increase in *on* time of at least 1 hour was higher in the opicapone 50 mg group than in the L‐dopa 100 mg group, but the differences were not statistically significant (*P* = 0.2384 and *P* = 0.3158, respectively; Table 2). Results from the other diary‐reported efficacy endpoints were in line with the results for the main efficacy endpoint and demonstrated that treatment with opicapone 50 mg resulted in a significantly greater reduction in the percentage of *off* time (*P* = 0.0015; Table 2), a greater increase in absolute *on* time (*P* = 0.0338; Fig. 1B, Table 2) and a greater percentage in *on* time compared with L‐dopa 100 mg (*P* = 0.0015; Table 2). Overall, there was an increase in *on* time without dyskinesia which was similar to the reduction reported for *off* time, with the increase being greater for the opicapone 50 mg group than for L‐dopa 100 mg group, although the difference was not statistically significant (64.8 min vs. 56.6 min; *P* = 0.6679; Fig. 1C, Table 2). This was accompanied by a small reduction in *on* time with troublesome dyskinesia, where the reduction was numerically higher for the opicapone 50 mg group than for the L‐dopa 100 mg group (−5.8 mins vs. −2.3 mins for L‐dopa 100 mg; *P* = 0.7721; Table 2). A higher proportion of patients in the opicapone 50 mg group than those in the L‐dopa 100 mg group showed improvements (minimally, much, or very much) from baseline to end of study treatment as assessed by CGI‐I and PGI‐C (Fig. 2, Table 2). Overall, both groups showed numerical improvements from baseline to end of study in MDS‐UPDRS (for the overall score and the scores for the individual Parts I–IV) and PDQ‐8 scores, but the differences between the groups were not significant (Table 2).

### Safety

No deaths occurred during the study. The percentage of patients who discontinued due to AEs was low (<5%) and similar between the treatment groups (Table 3). There was no common AE leading to discontinuation; all the five patients who discontinued due to AEs reported different reasons (spinal compression fracture, dizziness and rash in the opicapone 50 mg group; upper limb fracture and insomnia in the L‐dopa 100 mg group). Dyskinesia was the most frequently reported AE possibly related to the study drug, with the highest incidence observed in the opicapone 50 mg group (6 cases, 7%; Table 3). The incidence of serious AEs was low (<5%) and similar between the treatment groups; only 1 case was judged to be related to the study drug (Table 3).

**TABLE 3:** Summary of safety in the safety analysis set

	Opicapone 50 mg	L‐dopa 100 mg
	n = 87	n = 81
Any AE, n (%)^a^	33 (37.9)	15 (18.5)
Dizziness	8 (9.2)	3 (3.7)
Dyskinesia	7 (8.1)	1 (1.2)
Constipation	4 (4.6)	2 (2.5)
Headache	3 (3.5)	–
Asthenia	3 (3.5)	2 (2.5)
Severity, n (%)		
Mild	28 (32.2)	12 (14.8)
Moderate	6 (6.9)	3 (3.7)
Severe	1 (1.2)	–
Serious AEs, n (%)	3 (3.5)	1 (1.2)
Spinal compression fracture	2 (2.3)	–
Subdural hematoma	1 (1.2)	–
Upper limb fracture	–	1 (1.2)
AEs leading to discontinuation, n (%)	3 (3.5)	2 (2.5)
Spinal compression fracture	1 (1.2)	–
Upper limb fracture	–	1 (1.2)
Dizziness	1 (1.2)	‐
Insomnia	–	1 (1.2)
Rash	1 (1.2)	–
Any drug‐related AE, n (%)^a^	22 (25.3)	10 (12.4)
Dyskinesia	6 (6.9)	1 (1.2)
Dizziness	5 (5.8)	2 (2.5)
Constipation	4 (4.6)	2 (2.5)
Headache	3 (3.5)	–
Serious drug‐related AE, n (%)	1 (1.2)	–
Subdural hematoma	1 (1.2)	–

*Note*: n represents number of patients.

^a^ ≥3% of patients; AE, adverse event; L‐dopa, levodopa.

## Discussion

In this Phase 4 study, opicapone 50 mg was more efficacious than an additional L‐dopa dose of 100 mg in managing early wearing‐off symptoms. Opicapone 50 mg resulted in a significant additional mean reduction in *off* time of 45.4 mins versus L‐dopa 100 mg, which was accompanied by a corresponding non‐significant increase in *on* time without dyskinesia and a non‐significant decrease in *on* time with troublesome dyskinesia. The fact that changes in non‐motor symptoms (as assessed by the MDS‐UPDRS Part I), activities of daily living and quality of life were not significantly different between the two groups could reflect the relative low sensitivity to change of these measurements in patients with early wearing‐off treated with stable L‐dopa regimens. Furthermore, the study was not designed or powered to specifically evaluate these endpoints, as the majority of patients enrolled presented low scores at baseline and might not have shown improvements on these scales. Nevertheless, while we did not expect large treatment differences between opicapone 50 mg and L‐dopa 100 mg in scale‐based efficacy endpoints (ie, all patients were already receiving L‐dopa therapy and the comparator treatment was an additional L‐dopa dose), there was a trend for greater improvements in MDS‐UPDRS scores, quality of life (as assessed by the PDQ‐8), responder rates (for both *off* and *on* time) and the proportions of patients rated as improved by both investigators (CGI‐I) and patients (PGI‐C) for the opicapone 50 mg group compared with the L‐dopa 100 mg group.

Opicapone was safe and well tolerated. Dyskinesia was generally more common with opicapone 50 mg than with an additional L‐dopa dose of 100 mg. Nevertheless, as noted earlier, dyskinesias were deemed non‐troublesome by the patients. This finding is interesting and may well be related to the higher LEDD achieved with the addition of opicapone than with an additional dose of L‐dopa in this study population. *On* time with troublesome dyskinesia was reduced in the opicapone 50 mg while *on* time without dyskinesia was increased, suggesting an increase in peak‐dose non‐troublesome dyskinesia in this group, which might be related to improvements in fluctuations in plasma L‐dopa concentrations and reduced troughs. The incidence of serious AEs with opicapone 50 mg did not differ from that in the L‐dopa 100 mg group.

Since our LEDD calculations only result in a higher LEDD when a conversion factor of 0.5 is used (but not when a conversion factor of 0.33 is used), we suggest that a conversion factor of 0.5 could be more appropriate for opicapone in this study, as previously suggested by Schade et al.^20^

This study aimed to address the clinically relevant question of which strategies should be used to treat early wearing‐off symptoms, and, to our knowledge, it represents the only study comparing the addition of opicapone with an additional dose of L‐dopa. The high number of patients included in the study, the fact that only patients with early wearing‐off were recruited and that the majority of patients remained in the study, along with the inclusion of an active treatment arm of L‐dopa 100 mg are important strengths of the study. It should be noted that the results of the current study should not be directly compared with the results of the two pivotal clinical trials of opicapone (BIPARK‐I and‐II),^14^, ^15^ due to the different methodology (eg, duration of treatment and the fact that the current study evaluated the effects of two active interventions, with the comparator being L‐dopa itself) and differences in the population characteristics (patients in the BIPARK studies were from different countries and much more heterogenous, and presented with advanced PD [~8 years of disease duration; ~6 hours of time spent in *off* state per day; ~700 mg/day of L‐dopa]). It should also be considered that this trial compared the efficacy of opicapone add‐on therapy only with a 100‐mg fixed dose L‐dopa increment, and not with higher increments of L‐dopa doses. Because opicapone is thought to be equivalent to L‐dopa dose increase by 1.5 times, 100 mg add‐on might be too small in some patients. Nevertheless, it seems clear that opicapone add‐on is an effective option to improve early wearing‐off symptoms in PD patients, even for early morning off episodes.

Limitations of this study clearly include its exploratory nature, a single country population and the open‐label design. It is also important to notice that the additional dose of L‐dopa could have been taken in one full intake or split into two intakes and that the modality of administration might have affected the outcomes in the group of patients randomized to L‐dopa 100 mg. However, information on the modality of administration of the additional L‐dopa dose was not collected during the trial. This was an exploratory trial and further studies with a focus on direct comparisons between opicapone and different L‐dopa regimens and intake schemes are required to clarify the benefits of opicapone. It should be noted that this study concept (ADOPTION) is currently being undertaken in several countries in Europe (Portugal, Spain, Italy, Germany, and the UK).

This study complements the findings of a recent exploratory Phase 2 study of opicapone that highlighted the critical importance of opicapone in improving the pharmacokinetics of L‐dopa.^12^ The results suggested that combining opicapone 50 mg with a 100 mg lower daily dose of L‐dopa/carbidopa regimen significantly improved L‐dopa bioavailability by avoiding trough levels and, despite the lower L‐dopa dose, decreased *off* time in patients with PD and motor fluctuations. In line with these findings, the results of our current study indicate that opicapone is significantly more effective than an additional 100 mg dose of L‐dopa/DDCI to treat early wearing‐off. Taken together, these findings suggest that adding opicapone 50 mg to the existing L‐dopa/DDCI treatment might be more effective than adjusting the L‐dopa dose directly in the treatment of early wearing‐off, specifically by decreasing *off* time.

## Author Roles

(1) Research Project: A. Conception, B. Organization, C. Execution; (2) Statistical analysis: A. Design, B. Execution; C. Review and critique; (3) Manuscript Preparation: A. Writing of the First Draft, B. Review and Critique.

J‐Y.L.: 1B, 1C, 3A, 3B

H‐i.M.: 1B, 1C, 3A, 3B

B.J.: 1B, 1C, 3A, 3B

J.J.F.: 1A, 3A, 3B

J‐F.R.: 1A, 2A, 2C, 3A, 3B

Y.H.S.: 1B, 3B

I‐U.S.: 1B, 3B

T‐B.A.: 1B, 3B

D.Y.K.: 1B, 3B

S‐M.C.: 1B, 3B

J‐M.K.: 1B, 3B

C.S.L.: 1B, 3B

P.H.L.: 1B, 3B

J‐H.P.: 1B, 3B

J‐H.L.: 1B, 3B

M.Y.P.: 1B, 3B

S.J.K.: 1B, 3B

J.S.B.: 1B, 3B

S‐M.C.: 1B, 3B

H‐W.S.: 1B, 3B

H‐W.L.: 1B, 3B

S.Y.K.: 1B, 3B

## Disclosures

**Ethical Compliance Statement:** The clinical study protocol and the informed consent form were reviewed and approved by the respective independent ethics committee. The Institutional Review Boards were: Seoul Metropolitan Government‐Seoul National University, Boramae Medical Center, Hallym University Sacred Heart Hospital, Gachon University Gil Medical Center, The Catholic University of Korea St. Mary's Hospital, KyungHee University Medical Center, Korea University Ansan Hospital, Dong‐A University Hospital, Seoul National University Bundang Hospital, Asan Medical Center, Severance Hospital Human Research Protection Center, Soonchunhyang University Hospital Bucheon, Pusan National University Yangsan Hospital, Yeungnam University Hospital, Inje University Busan Paik Hospital, Inje University Sanggye Paik Hospital, Chung‐Ang University Hospital, Kyungpook National University Chilgok Hospital, Hallym University Dongtan Sacred Heart Hospital. All patients included in the study signed a patient consent form. All authors confirm that we have read the Journal's position on issues involved in ethical publication and affirm that this work is consistent with those guidelines.

**Funding Sources and Conflict of Interest:** The study, data collection/analysis/interpretation and editorial assistance are funded by Laboratórios Bial ‐ Bial ‐ Portela & Cª, S.A, Portugal and SK Chemicals (Gyeonggi‐do, Korea) Co., Ltd. Life Science Biz, Korea. The authors declare that there are no conflicts of interest relevant to this work.

**Financial Disclosures for the previous 12 months:** JYL received a research grant of NRF funded by the MSIT of Korea and a multidisciplinary research grant‐in‐aid and a focused clinical research grant‐in‐aid from the Seoul Metropolitan Government‐Seoul National University (SMG‐SNU) Boramae Medical Center, and speaker honorarium from Esai Korea, Bial, SK chemicals and Scientific Advisory Board of Regeners Inc. BJ has received research grant from Peptron and Abbvie Korea and has participated in an expert panel for AspenNeuroscience. JJF has received grants from GlaxoSmithKline, Grunenthal, Fundação MSD (Portugal), TEVA, MSD, Allergan, Novartis, Medtronic. Received consultancy and speaker fees and participated in advisory boards for GlaxoSmithKline, Novartis, TEVA, Lundbeck, Solvay, BIAL, Merck‐Serono, Merz, Ipsen, Biogen, Acadia, Allergan, Abbvie, Sunovion Pharmaceuticals, Zambon, Affiris and Angelini. J‐FR is an employee of Bial. The other authors declare nothing to disclose.

## Supporting information


**Figure S1.** Study design with timelines of study assessments. L‐dopa, levodopa; OPC, opicapone.
**Figure S2.** Patient disposition. ID, investigational drug; FAS, Full Analysis Set; L‐dopa, levodopa; PPS, Per Protocol Set.
