# Supplementary material for: Opicapone to Treat Early Wearing‐off in Parkinson's Disease Patients: The Korean ADOPTION Trial
Source: Mov Disord Clin Pract. 2024 Apr 9;11(6):655–65. doi: 10.1002/mdc3.14030 (PMC11145137; doi:10.1002/mdc3.14030)

# SUPPLEMENTARY INFORMATION

**Figure S1. Study design with timelines of study assessments.** L-dopa, levodopa; OPC, opicapone


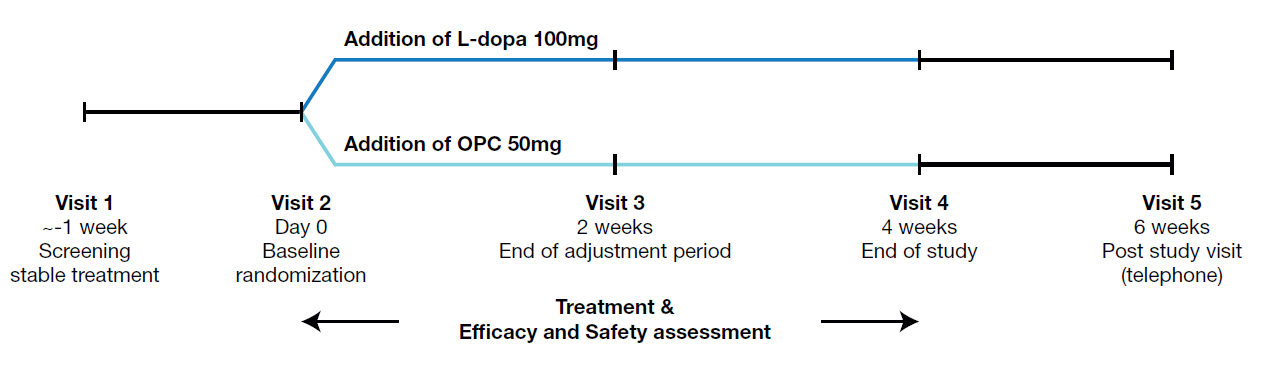


**Figure S2. Patient disposition.** ID, investigational drug; FAS, Full Analysis Set; L-dopa, levodopa; PPS, Per Protocol Set


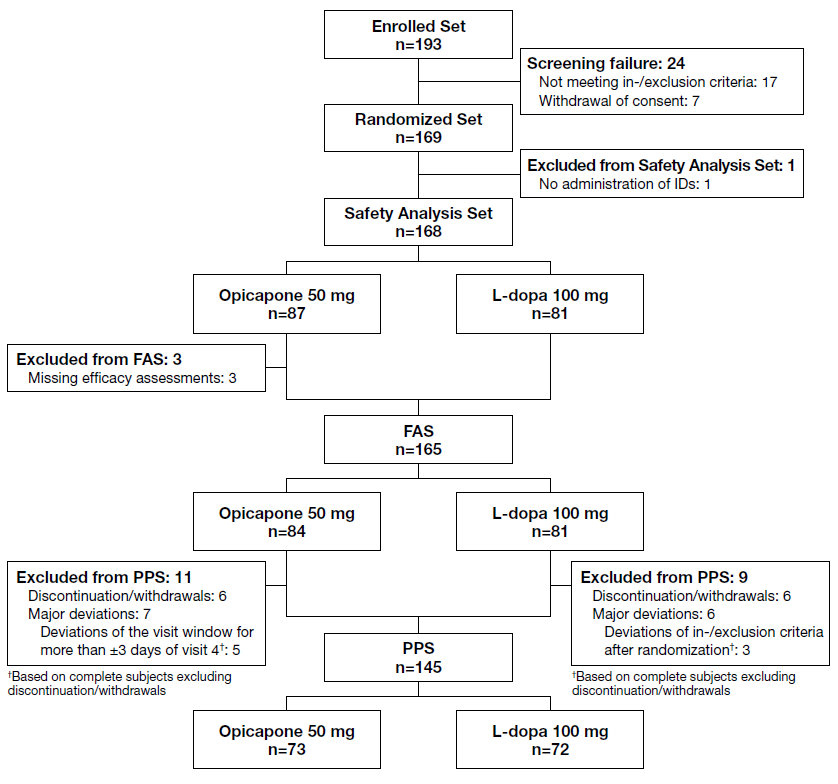

Supplement: Supplementary file 1 — Figure S1. Study design with timelines of study assessments. L‐dopa, levodopa; OPC, opicapone. Figure S2. Patient disposition. ID, investigational drug; FAS, Full Analysis Set; L‐dopa, levodopa; PPS, Per Protocol Set. [file MDC3-11-655-s001.docx]
